# Cross cultural adaptation and validation of the Hindi version of foot function index

**DOI:** 10.1186/s12998-024-00563-y

**Published:** 2024-12-05

**Authors:** Mohammad Sidiq, Aksh Chahal, Jyoti Sharma, Richa Hirendra Rai, Faizan Zaffar Kashoo, Jayaprakash Jayavelu, Neha Kashyap, Krishna Reddy Vajrala, T. S. Veeragoudhaman, Vinitha Arasu, Balamurugan Janakiraman

**Affiliations:** 1 Department of Physiotherapy, School of Allied Health Sciences, Galgotias University, Greater Noida, UP India; 2 School of Physiotherapy, Delhi Pharmaceutical Sciences and Research University, New Delhi, India; 3 Department of Physical Therapy and Health Rehabilitation, College of Applied Medical Sciences, Majmaah University, Al Majmaah, 11952 Saudi Arabia; 4 Department of Physical Therapy and Rehabilitation, Narayana Super Specialty Hospital, Gurugram, India; 5 Department of Physiotherapy, Maharishi Markandeshwar Deemed to Be University, Ambala, Haryana India; 6 SRM College of Physiotherapy, Faculty of Medicine and Health Sciences, SRM Institute of Science and Technology (SRMIST), Kattankulathur, Chennai, 603203 Tamil Nadu India

**Keywords:** Cross-cultural adaptation, Translation, Psychometric evaluation, Foot function index, Hindi, India

## Abstract

**Background:**

The Foot Function Index (FFI) is a reliable and widely used standardized questionnaire that measures the impact of foot pathology on function. With 571 million Hindi-speaking people living globally and an increasing incidence of foot-related pathologies, it is imperative to cross-culturally translate and adapt a Hindi version of the FFI (FFI-Hi). We aimed to translate, cross-cultural adapt, and psychometrically test the FFI-Hi for use in Hindi-speaking individuals with foot conditions.

**Methods:**

The translation of FFI-Hi was performed according to guidelines given by MAPI Research Trust. A total of 223 Hindi-speaking participants afflicted with foot conditions completed the FFI-Hi alongside the Short Form 36 (SF-36) questionnaire. The study duration spanned between October 2023 and January 2024. The initial phase was the translation and adaptation of FFI to cultural context. Followed by testing of psychometric properties involving of 133 participants for the test-retest reliability of FFI-Hi after a 7-day interval.

**Results:**

The mean age of the participants was 47.10 (± 8.1) years. The majority of the participants were male (*n* = 148, 66.4%) and the most common foot condition was plantar fasciopathy (*n* = 91, 40.8%). The mean score of FF-Hi was 33.7 ± 11.7. The internal consistency of FFI-Hi was good with the Cronbach’s alpha (α) value of 0.891 and excellent reproducibility with the intra-class correlation of 0.90. The 95% minimal detectable change (MCD) and the standard error of measurement of the FFI-Hi was 22.02 and 7.94 respectively. Convergent validity between FFI-Hi subscales and SF-36 domains was moderate. Factor analysis corroborated the multidimensional nature of the FFI-Hi.

**Conclusion:**

The FFI-Hindi version was successfully cross-culturally adapted, translated and demonstrated acceptable psychometric properties to be used in clinical practice and research. Further, the context-specific Hindi language version of FFI will enhance the utility of FFI in foot function evaluation and remove language barrier in patients reporting disability and activity limitation related to foot conditions.

**Registration:**

Clinical Trials Registry of India (CTRI/2023/07/055734).

**Supplementary Information:**

The online version contains supplementary material available at 10.1186/s12998-024-00563-y.

## Background

The global burden of musculoskeletal disorders is on the rise at an unprecedented rate [1]. The global estimates for 2019, reported that among the 2.41 billion individuals who live with diseases or conditions that would benefit from rehabilitation, about 1.71 billion of them suffer from musculoskeletal disorders [2]. Foot-related musculoskeletal disorders can be disabling and it is emerging to be a major public health concern, which is predicted to be escalated by factors like epidemic obesity, diabetes mellitus, work-related foot disorders, bone-related diseases, and aging [3–5]. The application of patient-reported outcome measures is vital in improving value-based clinical care, and it also enhances the clinical communication between patients and clinicians. Further, outcome measures provide better insight into the impact of treatment and the effect of their condition on their body, function, activity limitations, and restriction in participation [6, 7].

Although value-based patient care is often enhanced by the use of patient-reported outcome measures. Language and cultural barriers frequently obstruct patient-clinician communication and in most cases lower the standard of care [8]. Language barriers pose challenges in terms of achieving high levels of satisfaction among medical professionals and patients, providing high-quality healthcare, and maintaining patient safety [8]. The Foot Function Index (FFI) is a widely used self-reporting outcome measure designed to evaluate foot-related issues [9]. Comprising three domains with 23 items—pain (9 items), disability (9 items), and limitation of activity (5 items)—the FFI is valued for its feasibility, ease of use, and efficiency. Its multidimensional structure enables a comprehensive assessment of foot-related problems, rendering it pragmatic and time-saving tool in both clinical and research settings [10]. The FFI tool is designed for the assessment of perceived disability related to foot and ankle conditions. The psychometric properties of the FFI have been evaluated in patients with chronic ankle instability, plantar fasciitis, ankle arthritis, ankle sprain, and meta-tarsalgia. In addition, the FFI is translated for use in Arabic, Spanish, German, Chinese, and Thai versions [10–14]. The FFI is also used in studies to evaluate the effect of physical therapy, orthotic interventions, and surgery for various ankle and foot-related conditions [15–18]. Further, there are about 571 million Hindi-speaking people globally, and considering the projected escalation of foot disorders secondary to diabetes, obesity, bone density disorders, and aging-related degenerative disorders the need for reliable, relevant, accurate, and cultural context-based foot function index outcomes measure is warranted to improve healthcare delivery among people living with foot condition.

Despite its wide utility, there is no validated FFI tool available in the Hindi language. Therefore, this study aimed to cross-culturally adapt and translate the FFI questionnaire from its original version into Hindi language. Additionally, the study sought to assess the validity and reliability of the FFI-Hi among patients with foot conditions in India.

## Methods

This study was a cross-sectional design with the permission to cross-culturally adapt and translate the FFI was secured from MAPI Trust, a non-profit organization based in France and the copyright holder of the FFI, via a signed agreement (eprovide.mapi trust.org). Ethical clearance was obtained from the Departmental Ethics Committee, Galgotias University Greater Noida, India, and the research protocol was prospectively registered with the Clinical Trial Registry India under identifier CTRI/2023/07/055734 (dated June 2023). Before participation, all individuals were asked to give their informed consent by signing a consent form. Participants had the autonomy to withdraw from the study at any period without the need to provide reason of withdrawal.

### Sample size calculation

The study adhered to recommended best practices for developing and validating self-reported outcome tools, which advocate recruiting a minimum of 5 to 10 participants per item in the tool, as outlined in the COSMIN checklist [19]. Accordingly, this study aimed to include 230 participants (10 per item) to ensure the accuracy of the findings [20]. For sample size estimation, the study calculated the minimum power sample required for a 2-tailed Cronbach’s alpha test using the Bonett formula, with assumptions including 10 items in the outcome measure (k) 10, a power (1 - β) of 0.90, a type I error (α) of 0.05 (5% margin of error), and assumed values of 0.0 and 0.7 for Cronbach’s alpha at the null hypothesis (CA 0) and anticipated Cronbach’s alpha (CA 1) at 0.0 and 0.8, respectively [21]. This analysis determined that a sample size of *n* = 70 was required. Additionally, sample size estimation for the intra-class coefficient (ρ_1_) was conducted based on desired precision of 0.8, a 95% confidence interval, 2 repetitions per participant (test-retest), a 5% margin of error, and desired widths of 0.4 (ρ_0_ minimum acceptable ICC) to 0.7 (ρ_1_ expected reliability ICC) was *n* = 137 [21, 22]. Given the importance of robust psychometric testing, the study opted to use the higher estimate of 137 participants for the validation of the FFI-Hi.

### Participants and setting

Adult native Hindi speakers aged 18 and above with painful foot conditions secondary to degenerative and/or neuro-musculoskeletal disorders in the foot/ankle were invited to participate. The study involved 230 patients, with 151 males and 79 females using convenient sampling from the Department of Physiotherapy, Galgotias University Greater Noida, India, and the Department of Physiotherapy, Narayana Super Specialty Hospital, Gurugram, India representing different socio-demographic characteristics. Participants with a recent (six months) history of fracture, surgery, sensory disorders, vascular diseases, being pregnant, psychiatric disorders, diagnosed cancer, and diseases related to knee and/or hip and/or back region were excluded. The study duration spanned between October 2023 and January 2024. A subset of 133 participants completed FFI-Hi for the second time at an interval of 7 days for test-retest reliability measurements.

### Questionnaire

The questionnaires provided concise information about the study, incorporating details on inclusion and exclusion criteria, the consent form, and demographic data such as gender, age, weight, and height. Furthermore, participants were required to provide information on the affected foot, previous medical history, associated medical problems, use of any assistive devices, standing time, smoking habits, and the duration of the injury. Subsequently, all 230 participants completed both the translated Hindi version of the FFI questionnaire (FFI-Hi India) and the previously translated Hindi version of the Quality of Life Short Form 36 (SF-36) questionnaire [23, 24]. The questions within the FFI questionnaire are structured into three distinct subscales: pain, disability, and activity limitation [25]. Participants assigned a score to each question using a 0 to 10 visual analog scale, where a higher score denoted more severe pain or disability. The scores from each subscale were then totaled and expressed as percentages. The overall scores from the three subscales were combined and divided by 170 to yield the total average score. This approach facilitated the calculation of a comprehensive measure reflecting the participant’s overall experience of pain, disability, and activity limitation. The average time taken to complete the entire set of the questionnaire was 12 to 16 min. The higher FFI score indicates worse health status.

The SF-36 is a valid and reliable tool used to evaluate health-related outcomes in the Hindi-speaking Indian population [24]. Physical functioning (PF), role limitations due to physical health problems (RP), bodily pain (BP), social functioning (SF), general mental health covering psychological distress and well-being (MH), role limitations due to emotional problems (RE), vitality, energy, and fatigue (VT), and general health perceptions (GH) are the eight dimensions of health assessed by the 36 questions in the SF-36 questionnaire. This comprehensive instrument provides a multidimensional perspective on an individual’s health status. The total score from all domains was calculated and presented on a scale of 0 to 100, where higher scores indicate better quality of life. The decision to employ the SF-36 for assessing the convergent validity of the FFI (FFI-Hi) was grounded in several considerations. Firstly, the SF-36 is readily accessible in a validated Hindi version, facilitating its use in the target population. Secondly, the SF-36 measures a comparable construct related to health-related quality of life, enabling a meaningful comparison with the FFI. Finally, the widespread use of SF-36 in clinical settings enhances its relevance as a reference tool, providing a well-established benchmark for evaluating the construct (convergent and discriminant) validity of the FFI-Hi in the specific context of the study. A moderate to good correlation of FFI-Hi with the physical functioning and mobility domain of the SF-36 tool and VAS scale was hypothesized. Weak correlations with the emotional, mental function, and vitality domains of SF-36 were also expected. To assess the test-retest reliability of the FFI-Hindi India, participants were re-invited to complete the FFI-Hi questionnaire. This assessment helps to ensure that the FFI-Hindi India is stable over time when administered to the same individuals under similar conditions, reinforcing the reliability of the instrument.

### Translation and cross-cultural adaptation process

**Fig. 1:**
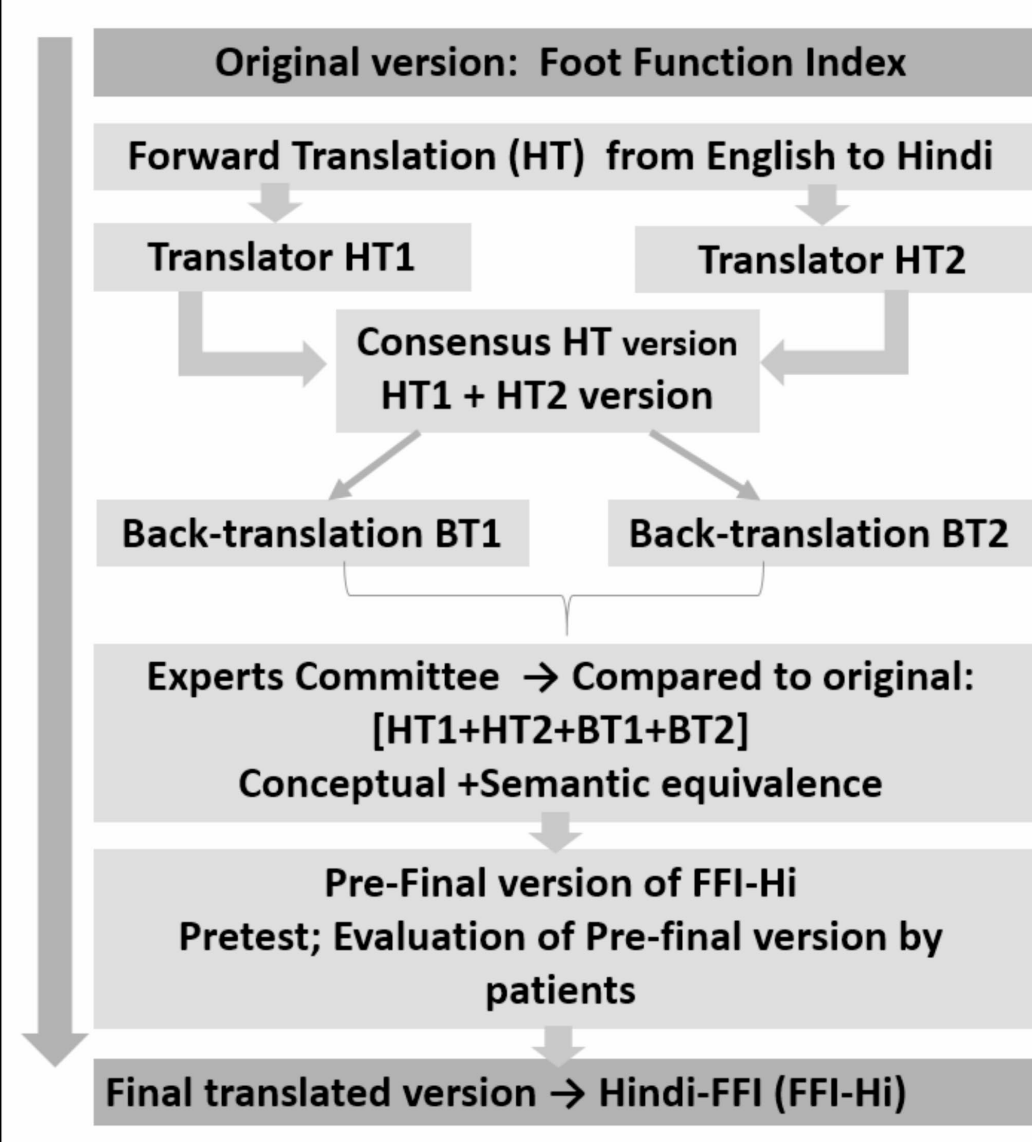
Flowchart of FFI-Hi questionnaire translation process

The process of translating and culturally adapting the FFI adhered to the methodology outlined by Beaton et al. [26]. This guideline encompasses six stages, comprising (1) forward translation, (2) synthesis, (3) backward translation, (4) expert community analysis, (5) pretesting, and (6) expert community evaluation of the entire process (Fig. 1).

In the initial step, Forward Translation, two bilingual native Hindi translators were engaged to translate the original English version of the FFI into a Hindi version. The initial translation of the original FFI version into Hindi was conducted by a knowledgeable translator with a background in physiotherapy (T1). T1, a native Hindi speaker fluent in English, brought a medical perspective to the translation. Additionally, a second translator without medical experience from an education background (T2) and with proficiency in both Hindi and English performed a blinded translation of the FFI into Hindi.

In the second step, Synthesis of the translations, the final translated version (T1,2) was derived by combining the translations from both T1 and T2. The translators (T1 and T2) along with a moderator engaged in discussions to review and reconcile any discrepancies between the two translated versions. Through this collaborative process, a unified and finalized version of the Hindi FFI (T1,2) was crafted, incorporating insights from both translators. During the third step, Back Translation, two bilingual back-translators produced the BT1 and BT2 versions. Fluent in Hindi and English, they independently translated the T12 version back into its original language, English. To minimize bias, the original version of the FFI was blinded from both back-translators, who spoke English as their primary language. After every back-translation procedure, each translator submitted a concise report. The committee members, including language experts, principal investigators, investigators, methodologists, and forward and back translators, gathered for the fourth step, the Expert Committee Review, to jointly assess all translated versions. The aim was to discuss, approve, and collaboratively formulate a pre-final version of the Hindi FFI.

In the fifth step, a Test of the Pre-final Hindi FFI Version was conducted through a pilot test involving 19 participants with a history of plantar fasciitis. The primary objectives were to assess the comprehensibility of the FFI questionnaire for all participants and gather their feedback and comments. Following the completion of the pre-final questionnaire by participants, their feedback was incorporated and documented. The mean time to complete the FFI-Hi was 5.65 (± 0.61) minutes. Only a few participants (*n* = 6) asked for clarifications regarding items 16 and 17 for the use of orthotics. In the sixth and final step, the Expert Committee convened to address and resolve all comments provided by the participants. Subsequently, they finalized the Hindi version of FFI, ensuring that the scale was ready for examination of its validity (Additional file 1). A healthy control of 40 subjects without foot and ankle problems also filled the FFI-Hi questionnaire and these subjects were recruited from the patient attenders visiting Narayana Super Specialty Hospital.

### Statistical analysis

Data were analyzed using the IBM Statistical Package for Social Science Version 21 for Windows (IBM SPSS INC, Chicago IL, USA).The descriptive statistics of the participants were expressed as mean, standard deviation, frequency, and percentage. The linear and normality hypotheses of the FFI-Hi scale were tested scatterplots, kurtosis (limit ± 2), skewness (limit ± 2), and Shapiro-Wilk’s test (*p* > 0.05).The intraclass-correlation coefficient (ICC _agreement_ 2,1) using a 2-way mixed effects model and Cronbach’s alpha (α) were estimated to assess the test-retest reliability and internal consistency of the FFI-Hi version, respectively. Cronbach’s alpha values of the FFI-Hi > 0.70 were considered acceptable, > 0.8 considered good, and > 0.9 considered excellent [27–29]. For test-retest reliability, Intraclass correlation coefficients (ICCs) and 95% confidence intervals (CIs) were determined. ICCs below 0.40 were considered low or unacceptable, those in the range of 0.4 to 0.70 were considered moderate, 0.70 to 0.90 were considered significant, and ‘α’ values above 0.9 were considered exceptional, respectively. The construct validity was considered to be low or weak if < 0.40, moderate or supportive if between 0.4 and 0.7, and good if < 0.7 [30, 31]. Reliability was also assessed using Mcdonald’s omega coefficient, with an adequate value being ω > 0.80 [32] **.** The Bland-Altman limit of agreement (LOA) for the 95% confidence interval was plotted to visualize the magnitude of the random changes by systematic variation or random measurement error [33].

Furthermore, the reliability of the FFI-Hi version was evaluated using the formulas for the minimum detectable change (MDC) = 1.96 √2x SEM and the standard error of measurement (SEM) = SD √ (1-R) [34]. The percentage of the respondent’s lowest and highest scores on the tool was used to determine the floor and ceiling effects of FFI-Hi. Assuming that the floor and ceiling effects did not surpass 15%, they were considered optimal [35]. The Exploratory factor analysis (EFA) was conducted to assess the theoretical and dimensional nature of the construct of FFI-Hi. The prerequisites were set using Kaiser-Meyer-Olkin coefficient (KMO), Eigen value of > 1,Barlett’s test of sphericity and visual inspection of scree plot. Parallel analysis was then conducted to determine the number of factors (Additional file 2, supplementary Table 2). The unweighted least squares method with oblimin rotation was employed for EFA of Hindi version of FFI-Hi and the minimum factor loading was set at 0.4 [36]. The underlying factor structure of the FFI-Hi items identified by the EFA was verified by conducting a confirmatory factor analysis (CFA) [37]. Confirmatory factor analysis (CFA) was conducted using JASP software version 0.19.1 for Windows. To examine the scale’s internal organization. As recommended for ordinal data, the robust weighted least squares estimator (WLSMV) was applied [38]. The indicators of the Goodness of Fit Index (GFI), Tucker-Lewis Index (TLI), Adjusted Goodness of Fit Index (AGFI), and Comparative Fit Index (CFI) were examined to confirm that the model was a good one; each should be greater than 0.95. A suitable fit is indicated by a value of less than 0.08 for the Root Mean Square Error of Approximation (RMSEA) and the Standardized Root Mean Square Residual (SRMR) [39]. A McDonald’s omega coefficient ꞷ was performed to assess the internal consistency [32]. There were no missing values in the dataset.

## Results

### Participants

The questionnaires with illogical responses (*n* = 7) were removed, and two hundred and twenty-three Hindi-speaking participants completed the FFI-Hi and SF-36 questionnaires. Among them, one hundred and thirty-three participants consented and completed the FFI-Hi again after 7 days to assess reliability. The mean age and body mass index (BMI) of the overall participants with painful foot conditions were 47.13 years and 25.47 kg/m^2^, respectively, and the majority of the participants were men (*n* = 148, 66.4%). The mean chronicity of the foot condition was 18 weeks (IQR 8, 36), and the majority reported right-side involvement (*n* = 132, 59.2%). Most participants suffered from plantar fasciopathy (40.8%), followed by meta-tarsalgia (24.2%), osteoarthritis (13.1%), rheumatic disease (5.8%), pes-planus (2.2%), and 13.9% reported foot pain with no particular clinical condition (Table 1). The characteristics of the 133 participants who completed FFI-Hi twice are shown in Table 1; the mean age was 45.64 years, 64.7% were men, and the most common foot condition was plantar fasciopathy (*n* = 56, 42.1%). When compared to patients with painful foot conditions (*n* = 233), the healthy controls (n 39) have significantly lower mean scores on the FFI-Hi subsets and total score (*p* < 0.0001 for all).

**Table 1:** Baseline data of the study participants

Variables	*n* = 223	^Ϯ^*n* = 133
Age (years): mean (± SD)	47.10 ± 8.1	45.64 ± 12.23
Male, n (%)	148 (66.4)	86 (64.7)
BMI (kg/m^2^); mean (± SD)	25.47 ± 1.66	25.7 ± 1.71
Duration of condition (weeks), median (IQR)	18.4 (8, 36)	20.4 (8, 36)
Foot orthosis, yes n (%)	52 (23.3)	32 (24.1)
Foot condition, n (%)		
Plantar fasciopathy	91 (40.8)	56 (42.1)
Metatarsalgia	54 (24.2)	34 (25.6)
Pes-planus	5 (2.2)	4 (3)
Rheumatic disease	13 (5.8)	6 (4.5)
Osteoarthritis	29 (13.1)	14 (10.5)
No pathology	31 (13.9)	19 (14.3)
Side involved, n (%)		
Right	132 (59.2)	80 (60.2)
Left	75 (33.6)	44 (33.1)
Both	16 (7.2)	9 (6.8)
VAS, day of inclusion:	5.12 ± 1.47	5.01 ± 1.30
VAS, past one week:	5.49 ± 1.35	5.45 ± 1.52

^Ϯ^Consecutive participants, VAS –Visual analog scale, IQR –Inter quartile range, SD – standard deviation

### Distribution of the FFI-Hi scores, floor and ceiling effects

The FFI-Hi scores for each item were plotted on a 10 cm horizontal line at the end of each question. An ‘NA’ option was available for each question, marked if the question was inconsistent with participants’ previous experiences, leading to an inability to rate the response to the item. The data were computed following recommended guidelines (E Budiman-Mak et al., 1991) and methodologies used in previous studies (Martinelli et al., 2014) (Wu et al., 2008). The maximum total score for FFI is 230. Scores for the three subscales and the overall FFI-Hi were converted to a scale of 0 to 100 using the formula (total score / 230) * 100. The mean and standard deviation of the overall participants’ FFI-Hi was 33.7 ± 11.7, with the means (SD) of the subscales for pain, disability, and activity limitation being 34.9 ± 13.2, 32.7 ± 15.9, and 33.6 ± 15.2, respectively.

The proportion of floor scores reported in the pain subscale ranged from 3 to 10 participants, while for the disability subscale, it ranged from 1 to 9, and for the activity limitation subscale, it ranged from 4 to 11 participants. Regarding ceiling scores, the proportion for the pain subscale ranged from 2 to 8 participants, for the disability subscale, it ranged from 3 to 13, and for the activity limitation subscale, it ranged from 3 to 8 participants. The mean, standard deviation, floor score, and ceiling score of each item are presented in Table 2.

**Table 2:** Descriptive statistics of FFI-Hi version, mean and standard deviation, and percentage of floor, and ceiling scores (*n* = 223)

Item	Mean ± SD	Floor score *n* (%)	Ceiling score *n* (%)
**Total score (0-100 scores)**	33.7 ± 11.7	-	-
**Pain subscale (0-100 scores)**	34.9 ± 13.2	-	-
*Sub-items 1–9 (0–10 scores)*			
1. Worst foot pain	3.5 ± 2.05	7 (3.1)	8 (3.6)
2. Morning foot pain	3.8 ± 2.26	14 (6.3)	5 (2.2)
3. Pain walking barefoot	3.7 ± 2.06	10 (4.5)	8 (3.6)
4. Pain standing barefoot	3.3 ± 2.19	4 (1.8)	8 (3.6)
5. Pain walking with shoes	3.4 ± 1.90	10 (4.5)	7 (3.1)
6. Pain standing with shoes	3.2 ± 1.56	5 (2.2)	5 (2.2)
7. Pain walking with orthotics	3.4 ± 1.58	3 (1.3)	5 (2.2)
8. Pain standing with orthotics	2.8 ± 1.91	7 (3.1)	2 (0.8)
9. Foot pain at end of day	3.9 ± 2.51	9 (4.0)	10 (4.5)
**Disability subscale (0 -100 scores)**	32.7 ± 15.9	-	-
*Sub-items 10–18 (0–10 scores)*			
10. Walking in house	3.7 ± 2.05	4 (1.8)	10 (4.5)
11. Walking outside	3.0 ± 2.06	0 (0)	8 (3.6)
12. Walking four blocks	3.5 ± 2.10	0 (0)	3 (1.3)
13. Climbing stairs	3.3 ± 2.24	0 (0)	9 (4.0)
14. Descending stairs	3.7 ± 2.48	1 (0.4)	13 (5.8)
15. Standing on tiptoes	2.7 ± 2.13	0 (0)	10 (4.5)
16. Getting up from chair	2.9 ± 205	4 (1.8)	9 (4)
17. Climbing curbs	3.3 ± 2.15	6 (2.7)	5 (2.2)
18. Running or walking fast	3.0 ± 2.24	9 (4)	3 (1.3)
**Activity limitation subscale (0-100 scores)**	33.6 ± 15.2	-	-
*Sub-items 19–23 (0–10 scores)*			
19. Using device indoors	2.8 ± 1.80	10 (4.5)	7 (3.1)
20. Using device outdoors	3.1 ± 2.32	11 (4.9)	8 (3.6)
21. Staying inside all day	3.5 ± 2.1	7 (3.1)	1 (0.4)
22. Staying in bed all day	3.5 ± 2.19	4 (1.8)	3 (1.3)
23. Limiting activities	3.7 ± 2.22	6 (2.7)	7 (3.1)

### Convergent validity between the FFI-Hi and short form 36 version (SF 36) questionnaire

The Spearman’s rank-order correlation coefficient test was employed to assess the convergent validity of the FFI-Hi, comparing it with the SF-36. The Spearman’s Rho correlation revealed that the scores of the three FFI-Hi subscales exhibited negative and moderate correlations with the eight domains of the SF-36. Specifically, the physical component of the SF-36, including BP, PF, and PR, showed good to moderate correlations with the overall FFI-Hi scores, with values of -0.70 (*rho*), -0.77 (*rho*), and − 0.59 (*rho*), respectively. In contrast, the mental components of the SF-36 displayed weak negative correlations with the overall FFI-Hi scores, with values of VT -0.40 (*rho*), SF 0.46 (*rho*), ER -0.45(*rho*), and MH -0.42(*rho*). Additionally, the pain and disability subscales of the FFI-Hi demonstrated high correlations with VAS intensity scores, with values of rho − 0.86 and rho − 0.71, respectively. Table 3 presents Spearman’s correlation coefficients for the FFI-Hi subscales, SF-36 domains, and VAS intensity during function.

**Table 3:** Spearman’s correlation of FFI-Hi with VAS and sub-scales of SF-36 (*n* = 133)

	Pain FFI-Hi	Disability FFI-Hi	Activity limitation FFI-Hi	FFI-Hi total
Bodily pain SF36	-0.531**	-0.69**	-0.44*	-0.70**
Physical functioning SF36	-0.67*	-0.85**	-0.72**	-0.77**
Social functioning SF36	-0.38*	-0.40*	-0.43*	-0.46**
Vitality SF36	-0.49*	-0.56**	-0.45*	-0.40*
Emotional role SF36	-0.47*	-0.35*	-0.41*	-0.45
Mental health SF36	-0.41*	-0.41*	-0.43**	-0.42*
Physical role SF36	-0.48*	-0.69**	-0.68**	-0.59**
General health SF36	-0.35*	-0.70**	-0.37	-0.38
VAS intensity	-0.86**	-0.71**	-0.43*	-0.65**

Spearman’s correlation (rho); low < 0.4, moderate 0.4 to 0.7, high > 0.7. Statistical significance (p values): * < 0.05, ** < 0.001. SF 36- short for, 36 questionnaire, VAS – Visual analogue scale

### Internal consistency and test-retest reliability of the FFI-Hi

**Fig. 2:**
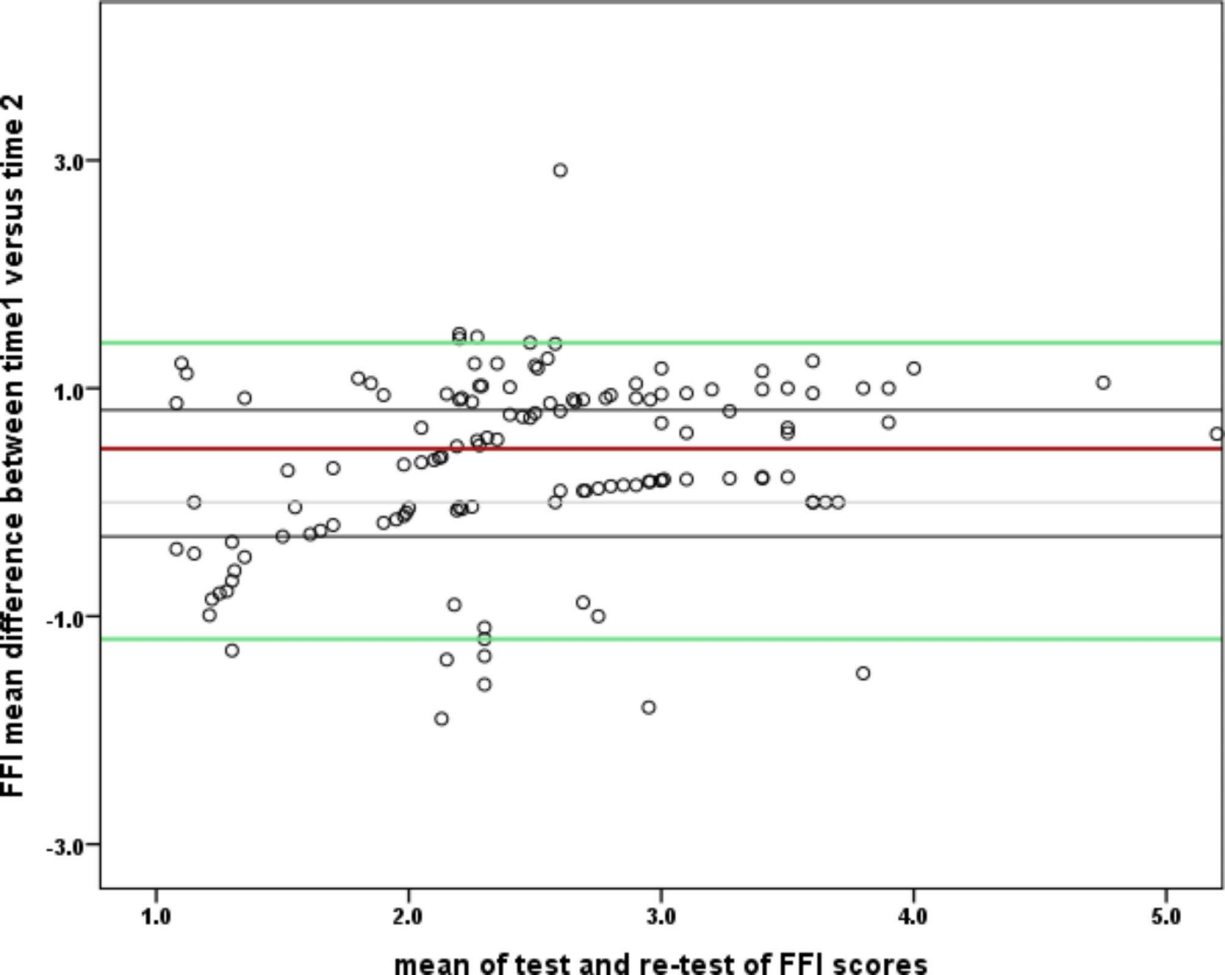
Bland Altman plot of agreement between the test and re-test scores of FFI-Hi. The bold red line represent the mean difference, the green lines represent the 95% limits of agreement (LOA), and the grey lines representing the 95% CI of the mean of the difference

The FFI-Hi exhibited excellent internal consistency, with a Cronbach’s alpha of 0.89 (Table 4). Furthermore, the three subscales of the FFI-Hi version also demonstrated good internal consistency, with values for pain (α 0.83), disability (α 0.85), and activity limitation (α 0.77). Analysis of internal consistency, with items deleted, yielded values ranging from 0.79 to 0.86, indicating consistent reliability even when certain items were removed (Table 5). The test-retest recordings of the FFI-Hi subscales showed excellent reliability: pain (ICC 3,1 = 0.86, 95% CI 0.73, 0.98), disability (ICC 3,1 = 0.91, 95% CI 0.81, 0.94), and activity limitation (ICC 3,1 = 0.80, 95% CI 0.69, 0.87). The overall test-retest reliability of the FFI-Hi was ICC 3,1 = 0.90, with a 95% CI of 0.81 to 0.96. To visually demonstrate the agreement between test-retest reliability, a Bland-Altman plot was generated (Fig. 2).

The plot illustrates that a significant proportion of observations fall within the 95% CI of the line of agreement (LOA). The overall standard error of measurement (SEM) and Minimal Detectable Change (MDC95) were 7.94 and 22.02 respectively (Table 4). The results of McDonald’s Omega coefficient showed high overall reliability (ω coefficient 0.91, 95% CI; 0.90, 0.94), and the item dropped McDonald’s Omega coefficient ranged from 0.912 to 0.918 (Additional file 2, Supplementary table 4a, 4b).

**Table 4:** Test-retest reliability of the FFI-Hi

FFI-Hi Subscales (items)	Mean (SD)		ICC (95% CI)	SEM	MCD 95%	Cronbach’s alpha α
	Test score	Retest score				
Pain(9)*	31.78 (11.6)	30.33 (11.1)	0.86 (0.73, 0.98)	4.24	11.74	0.831
Disability(9)*	30.22 (14.1)	27.83 (12.14)	0.91 (0.81, 0.94)	3.94	10.91	0.853
Activity limitation(5)*	17.18 (7.3)	14.96 (4.7)	0.80 (0.69, 0.87)	2.62	7.27	0.778
FFI-Hi(23)*	77.6 (26.4)	72.2 (23.3)	0.90 (0.81, 0.96)	7.64	22.02	0.891

**Table 5:** Internal consistency of FFI-Hi, if item deleted

Item total statistics		Scale mean if Item deleted	Scale variance if Item deleted	Corrected Item-total Correlation	Cronbach’s α if Item deleted
Subscale	Items				
Pain	1	27.94	113.13	0.56	0.81
	2	27.54	114.24	0.46	0.82
	3	27.67	116.07	0.48	0.82
	4	28.04	113.21	0.51	0.81
	5	28.06	115.32	0.56	0.81
	6	28.18	116.08	0.68	0.80
	7	28.02	117.46	0.63	0.81
	8	28.57	111.01	0.66	0.80
	9	27.48	111.93	0.44	0.83
Disability	10	25.66	170.61	0.61	0.89
	11	26.47	164.94	0.72	0.88
	12	25.93	170.02	0.62	0.89
	13	26.15	167.51	0.60	0.89
	14	25.68	167.55	0.53	0.89
	15	26.70	162.01	0.76	0.87
	16	26.57	162.12	0.79	0.87
	17	26.12	164.23	0.70	0.88
	18	26.46	166.30	0.63	0.88
Activity limitation	19	13.94	43.10	0.51	0.79
	20	13.72	38.86	0.49	0.78
	21	13.28	36.92	0.56	0.80
	22	13.29	37.69	0.60	0.79
	23	13.02	42.61	0.36	0.80

### Construct validity of FFI-Hi

**Fig. 3:**
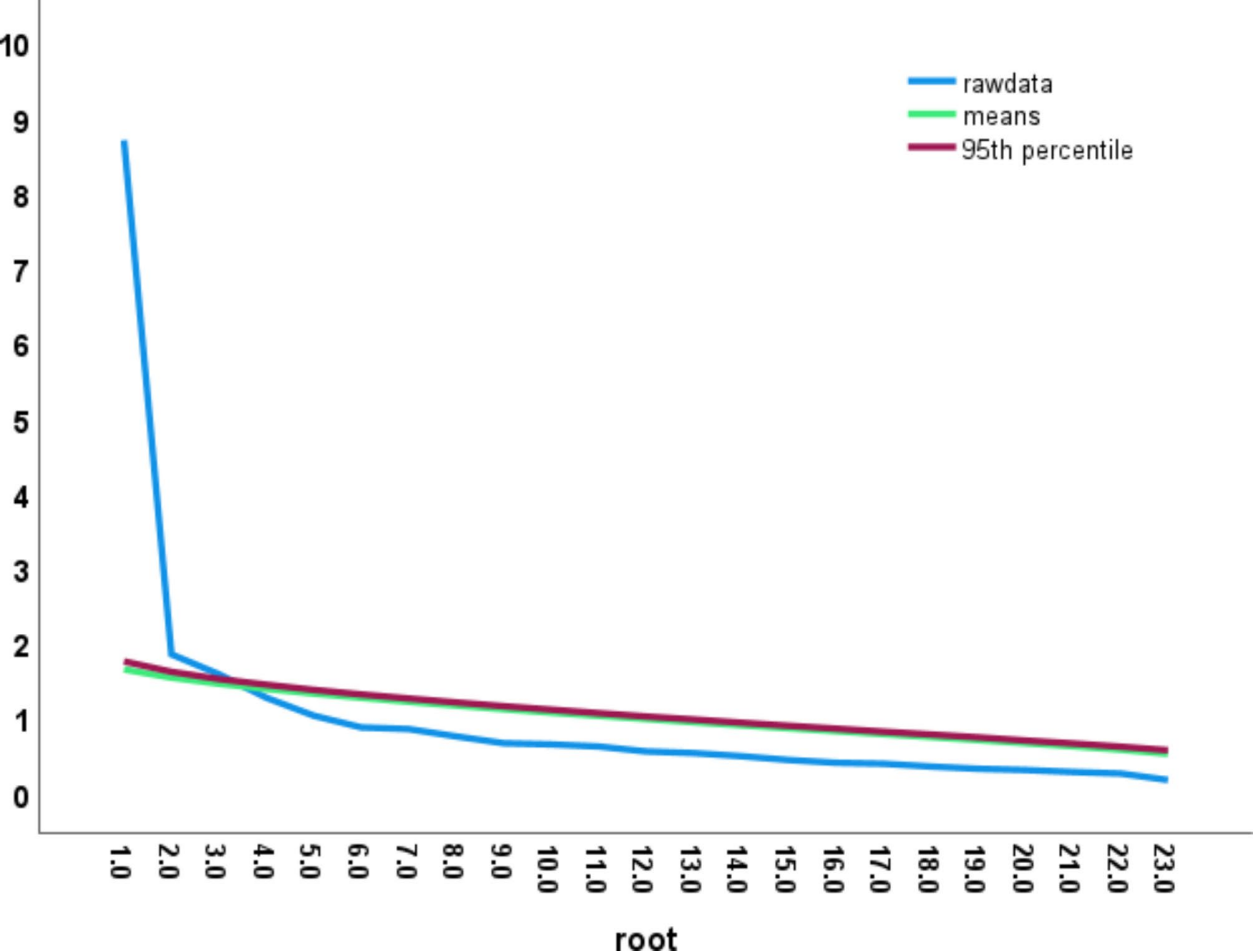
Scree plot indicating factor loading of FFI-Hi

The construct validity of FFI-Hi was estimated by principal component analysis using varimax rotation. The Kaiser-Meyer Olkin (KMO) of sample adequacy was assessed by Bartlett’s test of sphericity and found to be statistically significant (KMO 0.88, χ^2^ 2334.6, p value 0.01). The exploratory factor analysis grouped the 23 FFI-Hi items into five factors that explained 61.6% of the variance. The exploratory factor analysis grouped 23 items into 5 factors. (Table 6). Five-factor components had eigenvalues greater than 1 and the EFA scree plot (Fig. 3) and Parallel analysis (Additional file 2>, Supplementary Fig. 1) suggests the same.

**Fig. 4:**
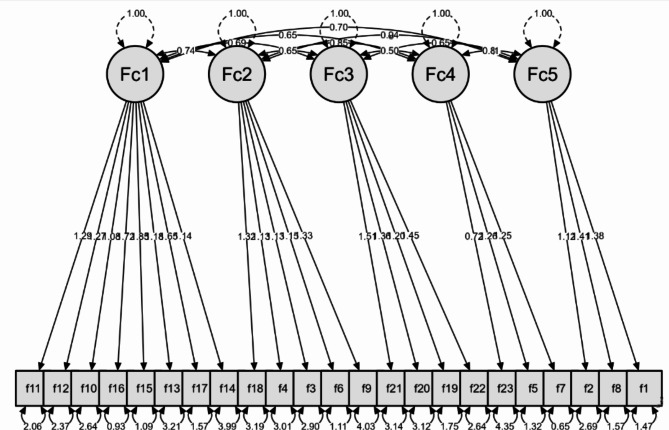
Path diagram of confirmatory factor analysis of 23-item 5 factor model (x^2^ = 2556.1, CFI = 0.87, TFL = 0.85, and RMSEA = 0.079)

The findings of CFA to examine the internal structure of the FFI-Hi scale indicated that the absolute goodness-of-fit was adequate (χ^2^ = 2556.13, df = 253, *p* < 0.001) and the additional goodness-of-fit indices (CFI = 0.87, TLI = 0.85, RMSEA = 0.079, and SRMR = 0.078) were satisfactory (Additional file 2, Supplementary Table 3). The five-factor internal structure of the FFI-Hi scale was confirmed by the CFA (Fig. 4) and the scale demonstrates an adequate level of validity and reliability.

### Discriminant validity of FFI-Hi

The results demonstrated that the healthy controls (*n* = 39) reported lower scores compared to the participants with painful foot conditions in the FFI-Hi version. The mean FFI-Hi score in healthy controls was 4.64 ± 2.01 (*n* = 39, 95% CI 1.76, 2.15) versus the FFI-Hi score in subjects with painful foot conditions 77.6 ± 27.1 (*n* = 233, 95% CI 74.2, 81.3). A statistically significant difference (*p* < 0.0001) was found between the foot pathology group and the healthy control regarding the total FFI-Hi score and all the three subsets of the scale (Additional file 2, Supplementary Table 1).

**Table 6:** Factor loading

	Factors				
Items	F1	F2	F3	F4	F5
Item #11	0.759				
Item #12	0.749				
Item #10	0.733				
Item #16	0.709				
Item #15	0.694				
Item #13	0.653				
Item #17	0.651				
Item #14	0.630				
Item #18		0.765			
Item #4		0.673			
Item #3		0.618			
Item #6		0.588			
Item #9		0.510			
Item #21			0.735		
Item #20			0.711		
Item #19			0.693		
Item #22			0.659		
Item #23				0.799	
Item #5				0.725	
Item #7				0.501	
Item #2					0.474
Item #8					0.428
Item #1					0.402
% of Variance explained	32.9	10.46	8.24	5.6	4.47

Kaiser Meyer Olkin measure (KMO) = 0.880, Chi-square χ^2^ = 2343.4, Extraction method: Unweighted Least Squares, Rotation method: Oblimin with Kaiser Normalization

## Discussion

The successful cross-cultural adaptation and translation of the FFI into Hindi, followed by the subsequent evaluation of its psychometric properties among individuals with different foot conditions in Northern India, will pave the way to significant advancements in foot and ankle research, particularly concerning patient-reported outcome measures (PROMs).

### Cross-cultural adaptation and translation of the FFI-Hi

The development of the Hindi version of the FFI (FFI-Hi) underwent a meticulous translation and adaptation process, following established guidelines. This rigorous methodological approach ensured the linguistic and conceptual equivalence of the translated instrument, thereby enhancing its applicability and relevance in the target population. By incorporating input from bilingual experts and conducting pilot testing with PF patients, the study ensured the comprehensibility and cultural appropriateness of the FFI-Hi, thus improving its validity and reliability. In the first phase of forward translation, modifications were made to two questions (5 and 6) to better align with Hindi culture and environment, considering that not all individuals wear shoes; many prefer chappals (slippers) instead. In the second phase, although no translated questions were rejected by the panel, some linguistic and grammatical corrections were suggested. Backward translation confirmed that both versions maintained the same conceptual meaning.

### Internal consistency of FFI-Hi

The evaluation of the psychometric properties of the FFI-Hi yielded promising results, affirming its utility as a valid and reliable instrument for assessing foot function among Hindi-speaking individuals with PF. The FFI-Hi demonstrated good internal consistency, indicating the homogeneity of items within each subscale. Similarly, a study aimed to validate the French version of the FFI to assess rheumatoid foot in French-speaking populations involving 53 patients with rheumatoid arthritis [40]. Internal consistency (Cronbach’s alpha: 0.85–0.97) was robust, with satisfactory reproducibility, good external validity, and responsiveness to change [40].

A study that validated the Spanish version of the Foot Function Index (FFI-Sp) reported high internal consistency across its subscales (pain: α = 0.95, disability: α = 0.96, activity limitation: α = 0.69) [11]. Another study assessing the reliability and validity of the Korean version of the FFI demonstrated high internal consistency (Cronbach’s α: pain = 0.91, disability = 0.95) [41]. Similar to our patient population, a Brazilian Portuguese version of the FFI demonstrated ICC ranging from 0.97 to 0.99 [42]. Similarly, a Taiwan-Chinese version of the FFI demonstrated high internal consistency (Cronbach’s α = 0.94) among patients with plantar fasciitis and ankle/foot fracture [43].

### Test-retest reliability of the FFI-Hi

Additionally, the high test-retest reliability of the FFI-Hi suggests its stability over time, reinforcing confidence in its reproducibility and consistency in measuring foot function among PF patients. A study reported that the FFI-Revised was translated into Turkish and administered to 124 patients, demonstrating high test-retest reliability (0.84–0.97) and internal consistency (0.97 overall, 0.85–0.97 for subscales) [44]. Similarly, Taiwan–Chinese version of the FFI demonstrated satisfactory test-retest reliability (ICC = 0.82) among patients with plantar fasciitis and ankle/foot fracture [43]. Another study assessing the reliability and validity of the Korean version of the FFI reported good test-retest reliability [41].

### Convergent validity of the FFI-Hi

Convergent validity analysis provided further support for the FFI-Hi, revealing low to moderate correlations between its subscales (Pain, Disability, and Activity Limitation) with scores on the VAS and subscales of the Short Form 36 (SF-36) questionnaire. The strongest correlations were observed with physical functioning SF-36 (-0.67 to -0.85) and VAS intensity (-0.43 to -0.86), indicating that foot-related issues significantly impact physical function and pain intensity during functional activities. This finding suggests that the FFI-Hi effectively captures aspects of foot-related issues aligned with broader domains of health-related quality of life assessed by the SF-36. The observed correlations validate the FFI-Hi as a relevant tool for evaluating foot function within the context of overall health and well-being among PF patients. Similarly, a Brazilian Portuguese version of the Foot Function Index (FFI) demonstrated strong validity, with correlations between FFI and SF-36 “pain” (*r* = 0.65) and “social aspects” (*r* = 0.59) subscales, as well as all FAOS subscales (r ranging from 0.54 to 0.73) (Martinez et al., 2016). Another study validating the Spanish version of the Foot Function Index (FFI-Sp) through a cross-sectional analysis with 80 participants reported strong correlations with related questionnaires, such as the Foot Health Status Questionnaire, EuroQol 5-D, Visual Analogue Pain Scale, and the Short Form SF-12 Health Survey [11].

### Construct validity of FFI-Hi

Furthermore, factor analysis confirmed the multidimensional structure of the FFI-Hi, consistent with its original design. The distinct subscales measuring pain, disability, and activity limitation exhibited appropriate factor loadings, supporting the conceptual integrity of the instrument. The robust factorial structure of the FFI-Hi enhances its sensitivity in detecting various dimensions of foot dysfunction among Hindi-speaking individuals with PF, facilitating a comprehensive assessment of their functional status. The multidimensionality of the scale was also observed in the original English version of the FFI scale [25].

Overall, the successful adaptation and validation of the FFI into Hindi represent a significant contribution to the assessment of foot function in diverse cultural and linguistic contexts. The availability of a validated instrument tailored to the Hindi-speaking population expands opportunities for clinical research, patient care, and intervention monitoring in India and other regions with Hindi-speaking communities. The FFI-Hi holds promise for enhancing the understanding of PF-related outcomes and informing evidence-based interventions to improve the quality of life for affected individuals.

### Floor and ceiling effect

The study observed limited floor effects across the subscales, with percentages ranging from 0 to 11%. This suggests that while some participants reported minimal difficulty or impairment in specific areas, the majority of responses covered a wider range, indicating the scale’s sensitivity in capturing diverse experiences. Conversely, ceiling effects were more evident, particularly within the disability subscale, where percentages ranged from 1.3 to 13%. This implies that a notable proportion of participants reported experiencing the highest levels of disability in certain activities, potentially indicating limitations in the scale’s ability to fully capture impairment in these areas. While interpreting the findings of this study, it’s essential to consider some limitations. These include the lack of analysis of longitudinal psychometric variables reporting the sensitivity to change when using FFI-Hi. However, the use of a power-calculated sample based on literature recommendations would enhance the accuracy of the findings.

### Future development, extensions, and implication

Future research endeavors should prioritize the extensive validation of the FFI-Hi across larger and more diverse samples. Additionally, exploring its responsiveness to clinical interventions and its predictive validity in longitudinal studies would provide valuable insights. A systematic review highlighted that the older version of the Foot Function Index (FFI) with five categories demonstrated better usability and friendlier language compared to the version with six categories [45]. Similarly, a study suggested that adding a psychosocial scale further enhanced the person and item reliability of FFI [46]. The FFI-Hi can be used in clinical and research settings to measure foot function-related disability and its impact among the Hindi-speaking people living with foot problems. Considering the lower rate of English proficiency and non-native English language speakers in India, the FFI-Hi would surely help scientific communities and clinicians overcome the language barrier to promote equity in science.

## Conclusion

In conclusion, the successful adaptation of the Foot Function Index (FFI) into Hindi (FFI-Hi) represents a significant stride in foot and ankle research, particularly for patients grappling with painful and disabling foot conditions in Northern India. The meticulous translation processes ensured the linguistic and conceptual equivalence of the FFI-Hi, rendering it pertinent for the Hindi-speaking populace. Psychometric evaluations affirmed its validity and reliability in assessing foot function, boasting good internal consistency and high test-retest reliability. Moreover, the FFI-Hi exhibited convergent validity by correlating with related measures such as pain intensity and SF-36 subscales, while factor analysis confirmed its multidimensional structure. However, the observed ceiling effects in the disability subscale imply the necessity for further refinement. Moving forward, future research should validate the FFI-Hi in diverse populations and explore its responsiveness to interventions. Ultimately, the FFI-Hi holds the potential to elevate clinical research and enhance patient care within Hindi-speaking communities affected by foot conditions.

## Electronic supplementary material

Below is the link to the electronic supplementary material.

Supplementary Material 1

Supplementary Material 2

Supplementary Material 3

Supplementary Material 4

Supplementary Material 5

Supplementary Material 6

## Data Availability

No datasets were generated or analysed during the current study.

## Abbreviations

FFI: Foot function index
CFA: Confirmatory factor analysis
EFA: Exploratory factor analysis
KMO: Kaiser–Meyer–Olkin
CFI: Comparative fit index
TLI: Tucker–Lewis index
GFI: Goodness of fit index
PA: Parallel analysis
RMSEA: Root mean square error of approximation
SRMR: Standardized root mean square residual
