# Supplementary material for: Cross cultural adaptation and validation of the Hindi version of foot function index
Source: Chiropr Man Therap. 2024 Dec 5;32:38. doi: 10.1186/s12998-024-00563-y (PMC11619674; doi:10.1186/s12998-024-00563-y)
Supplement: Supplementary file 1 — Supplementary Material 1 [file 12998_2024_563_MOESM1_ESM.pdf]

Title : Cross Cultural adaptation, Translation and Psychometric evaluation of the Hindi version of Foot Function Index

Full Process of the Steps

Foot Function Index (FFI) Tool adaptation and translation was conducted as per the guidelines suggested by Beaton et, al 2000 (Beaton et al. 2000). The translation and adaptation process for FFI consists of the following steps; forward translation, synthesis, backward translation, expert committee review, and synthesis, tool pretesting, and submission and appraisal of the written reports by the translation-adaptation coordinating committee(Beaton et al. 2000). In step 1, forward translation was performed from English to Hindi FFI language by two Hindi FFI native translators (a professional translator and a post-graduate employee working in education department) who were fluent in the English language. One independent translator was blinded to process of forward translation of FFI. This was done to make sure the equivalency from a therapeutic point of view rather than literal equivalence. The other translator was informed about the purpose of the study and the concepts being studied. This was for contemplating the language used by the population and spotlight terms in the original questionnaire, the translation of which might have been obscure. Two forward-translated documents (T1 and T2) were produced. In step 2, the independently translated T1 & T2 documents were shared among the 2 translators to synthesize FFI-Hi (version 1). Any inconsistencies, differences in the concepts and/or meaning, elusive wordings were sorted by discussion and agreement. In step 3, the 1st draft FFI-Hi will be back-translated to English language by two bilingual independent translators (native Hindi FFI language speaking professors with Doctoral degree in English) and not familiar with the construct being assessed and blinded to the process of forward translation. After discussion and agreement between back translators and the principal investigator (MS), a third translation will be performed. In step 4, all 3 documents will be presented to the expert panel for review and discussion. The expert committee will comprise of 4 translators, 2 review committee experts from the university and 2 senior physiotherapy academicians and 1 Orthopaedic surgeon who will review the Hindi FFI version of the questionnaire. The panel will discus on the clarity, relevance, modifications, comprehension and synthesized the pre-final version for field testing before a pilot study on fifteen patients with ankle of foot pain can be commenced.

Figure 1: Study flowchart

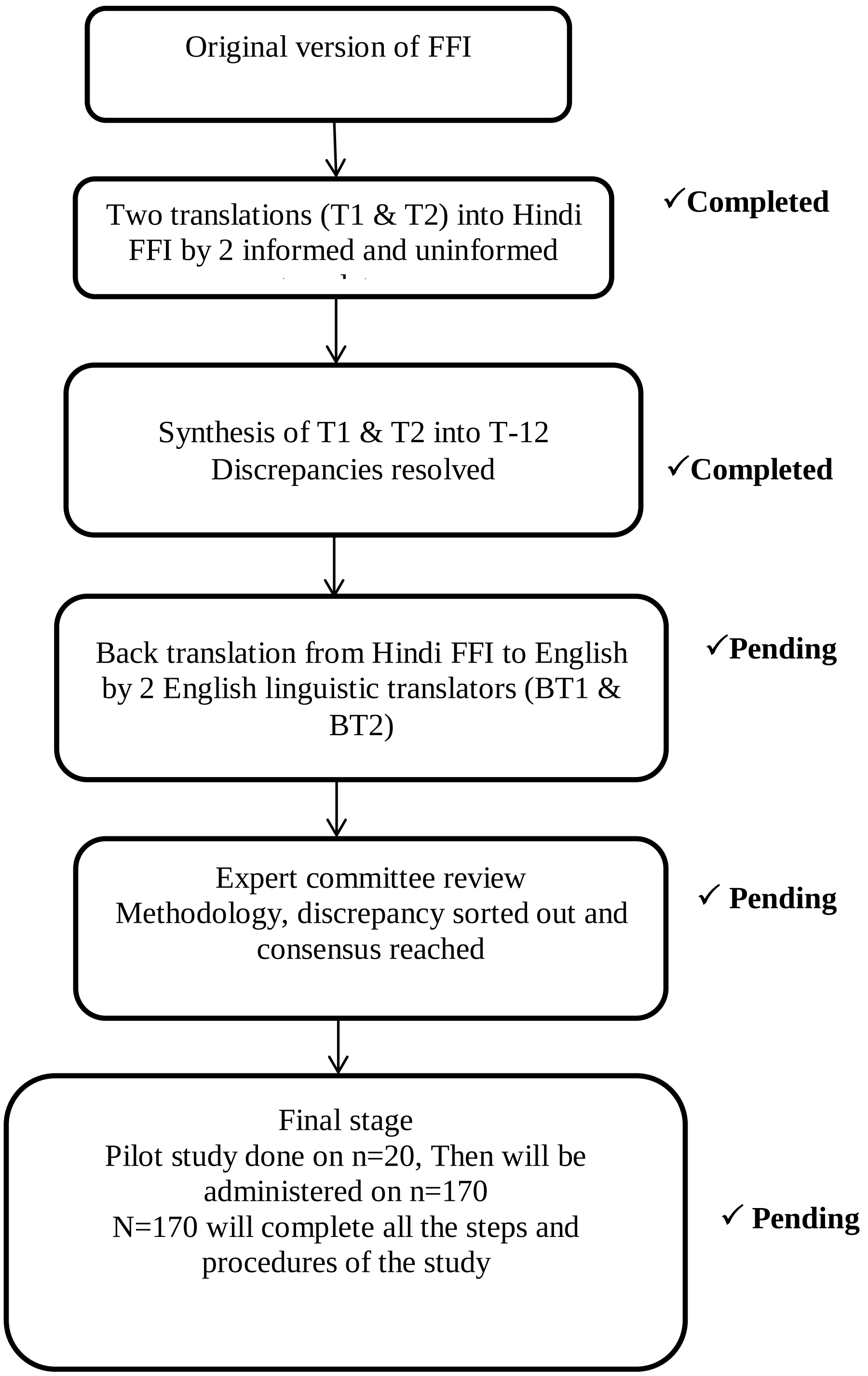

Bibliography :

Beaton, Dorcas E, Claire Bombardier, Francis Guillemin, and Marcos Bosi Ferraz. 2000. “Guidelines for the Process of Cross-Cultural Adaptation of Self-Report Measures.” 25(24): 3186–91.
Beaton, Dorcas E, Claire Bombardier, Francis Guillemin, and Marcos Bosi Ferraz. 2000. “Guidelines for the Process of Cross-Cultural Adaptation of Self-Report Measures.” 25(24): 3186–91.
