# Supplementary material for: Cross cultural adaptation and validation of the Hindi version of foot function index
Source: Chiropr Man Therap. 2024 Dec 5;32:38. doi: 10.1186/s12998-024-00563-y (PMC11619674; doi:10.1186/s12998-024-00563-y)
Supplement: Supplementary file 3 — Supplementary Material 3 [file 12998_2024_563_MOESM3_ESM.pdf]

Clinical Trial Details (PDF Generation Date :- Wed, 26 Jul 2023 09:05:31 GMT)

**CTRI Number**	CTRI/2023/07/055734 [Registered on: 26/07/2023] - **Trial Registered Prospectively**
**Last Modified On**	13/07/2023
**Post Graduate Thesis**	No
**Type of Trial**	Observational
**Type of Study**	Cross Sectional Study
**Study Design**	Other
**Public Title of Study**	"Development and Evaluation of the Hindi Version of the Foot Functional Index"
**Scientific Title of Study**	Cross cultural Adaptation, Translation and Psychometric Evaluation of the Hindi Version of Foot Functional Index

Secondary IDs if Any

Secondary ID	Identifier
NIL	NIL

Details of Principal Investigator or overall Trial Coordinator (multi-center study)

Details of Principal Investigator	
**Name**	Mohammad Sidiq
**Designation**	Associate Professor
**Affiliation**	Galgotias University
**Address**	Room No 316, Department of Physiotherapy, Plot No2, Sector 17A, Opposite Yamuna Expressway, Greater Noida, Gautam Buddha Nagar<br>Gautam Buddha Nagar<br>UTTAR PRADESH<br>203201<br>India
**Phone**	6006204628
**Fax**	
**Email**	sidufatima@gmail.com

Details Contact Person (Scientific Query)

Details Contact Person (Scientific Query)	
**Name**	Mohammad Sidiq
**Designation**	Associate Professor
**Affiliation**	Galgotias University
**Address**	Room No 316, Department of Physiotherapy, Plot No2, Sector 17A, Opposite Yamuna Expressway, Greater Noida, Gautam Buddha Nagar<br><br>UTTAR PRADESH<br>203201<br>India
**Phone**	6006204628
**Fax**	
**Email**	sidufatima@gmail.com

Details Contact Person (Public Query)

Details Contact Person (Public Query)	
**Name**	Professor Aksh Chahal
**Designation**	Professor
**Affiliation**	Galgotias University
**Address**	Room No 512A, Department of Physiotherapy, Plot No2, Sector 17A, Opposite Yamuna Expressway, Greater Noida, Gautam Buddha Nagar<br>Gautam Buddha Nagar<br>UTTAR PRADESH<br>203201

	India
**Phone**	9711774174
**Fax**	
**Email**	drakshchahal@gmail.com

Source of Monetary or Material Support

Source of Monetary or Material Support
> Research and Development Cell, Galgotias University

Primary Sponsor

Primary Sponsor Details	
**Name**	Galgotias University
**Address**	Plot No2, Sector 17A, opposite Gautam Buddha International circuit, Yamuna expressway, Greater Noida
**Type of Sponsor**	Research institution

Details of Secondary Sponsor

Name	Address
NIL	NIL

Countries of Recruitment

List of Countries
India

Sites of Study

Name of Principal Investigator	Name of Site	Site Address	Phone/Fax/Email
Prof Dr Aksh Chahal	Room No E003, Department of Physiotherapy, SMAS, Galgotias University	Plot No 2, Sector 17A, Opposite Gautam Buddha International Circuit, Yamuna expressway, Gautam Buddha Nagar UTTAR PRADESH	9711774174<br><br>drakshchahal@gmail.com

Details of Ethics Committee

Name of Committee	Approval Status	Date of Approval	Is Independent Ethics Committee?
Departmental Ethics Committee	Approved	10/07/2023	Yes

Regulatory Clearance Status from DCGI

Status	Date
Not Applicable	No Date Specified

Health Condition / Problems Studied

Health Type	Condition
Patients	Unspecified soft tissue disorder related to use, overuse and pressure

Intervention / Comparator Agent

Type	Name	Details
Intervention	NA	NA
Comparator Agent	NA	NA

Inclusion Criteria

Inclusion Criteria	
**Age From**	18.00 Year(s)
**Age To**	60.00 Year(s)
**Gender**	Both
**Details**	1. Language proficiency: Participants should be native Hindi speakers or individuals fluent in Hindi, as they will be required to understand and respond to the translated FFI questionnaire.<br>2. Age range: Specify the age range that is relevant to your study. For example, participants could be adults aged 18 to 65 years or a specific subgroup within that range.<br>3. Foot-related condition: Include individuals who have a foot-related condition or injury that affects their foot function. This could include conditions such as plantar fasciitis, ankle sprains, or other foot-related problems.<br>4. Clinical diagnosis: Participants should have received a clinical

	diagnosis of the foot-related condition by a qualified healthcare professional. This ensures that participants have a confirmed condition that can be assessed using the FFI.<br>5. Willingness to participate: Participants should be willing to take part in the study and comply with the study procedures, including completing the translated FFI questionnaire and any follow-up assessments or interviews.<br>6. Ability to provide informed consent: Participants should have the capacity to understand the study purpose, procedures, and potential risks involved. They should be able to provide informed consent or have a legally authorized representative who can provide consent on their behalf.

Exclusion Criteria

Exclusion Criteria	
**Details**	1. Inability to understand or communicate in Hindi: Participants who do not have sufficient proficiency in Hindi to understand the translated FFI questionnaire or provide meaningful responses should be excluded.<br>2. Cognitive impairment: Individuals with severe cognitive impairment or neurological conditions that impair their ability to comprehend and respond to the questionnaire accurately may be excluded.<br>3. Medical contraindications: Exclude individuals who have medical conditions or physical limitations that prevent them from completing the FFI questionnaire or participating in the study procedures safely. For example, individuals with severe foot deformities or amputations that significantly affect foot function may be excluded.<br>4. Participation in a similar study: Exclude individuals who have participated in a similar cross-cultural adaptation or translation study of the FFI questionnaire to avoid duplicating data or potential bias from prior exposure to the instrument.<br>5. Concurrent participation in another research study: Exclude individuals who are currently participating in another research study that may interfere with their ability to fully engage in the pilot study.<br>6. Inability to provide informed consent: Exclude individuals who lack the capacity to understand the purpose, risks, and procedures of the study and cannot provide informed consent, or who do not have a legally authorized representative available to provide consent on their behalf.

**Method of Generating Random Sequence**	Not Applicable
**Method of Concealment**	Not Applicable
**Blinding/Masking**	Not Applicable

Primary Outcome

Outcome	Timepoints
Pain<br>Disability<br>Foot Functional Index	Baseline & forty eight Hours later test-retest reliability

Secondary Outcome

Outcome	Timepoints
Range of Motion of Ankle & Subtalar Joint	Baseline & seventy two Hours

**Target Sample Size**	**Total Sample Size=**85<br>**Sample Size from India=**85<br>**Final Enrollment numbers achieved (Total)=**Applicable only for Completed/Terminated trials<br>**Final Enrollment numbers achieved (India)=**Applicable only for Completed/Terminated trials
**Phase of Trial**	N/A
**Date of First Enrollment (India)**	10/08/2023
**Date of First**	No Date Specified

**Enrollment (Global)**	
**Estimated Duration of Trial**	**Years=**0<br>**Months=**2<br>**Days=**10
**Recruitment Status of Trial (Global)**	Not Yet Recruiting
**Recruitment Status of Trial (India)**	Not Yet Recruiting
**Publication Details**	N/A
**Brief Summary**	Title: Cross-Cultural Adaptation and Translation of the Hindi Version of Foot Functional Index: A Pilot Study<br><br>Introduction:<br><br>The Foot Functional Index (FFI) is a widely used questionnaire designed to assess foot-related functional limitations and pain in individuals with foot conditions. However, to ensure its applicability in different cultural contexts, it is crucial to conduct cross-cultural adaptation and translation studies. This summary presents the key aspects of a pilot study focused on the cross-cultural adaptation and translation of the Hindi version of the FFI.<br><br>Objective:<br><br>The primary objective of this pilot study is to adapt and translate the FFI into Hindi, the native language of a significant population in India, and evaluate its comprehensibility, acceptability, and relevance in the Hindi-speaking population.<br><br>Methods:<br><br>The study will follow established guidelines for cross-cultural adaptation and translation of questionnaires. Initially, the FFI will undergo forward translation from English to Hindi by two independent translators, both proficient in the target language. Subsequently, a reconciliation process will be conducted to resolve any discrepancies and create a harmonized version of the translated questionnaire. Back-translation will then be performed by two other translators who are fluent in English but unfamiliar with the original FFI. A committee consisting of experts, including healthcare professionals and linguists, will review all versions to ensure semantic, idiomatic, and conceptual equivalence.<br><br>Participants:<br><br>Participants will be recruited from healthcare facilities and community settings in Hindi-speaking regions. Inclusion criteria will encompass native Hindi speakers or individuals fluent in Hindi, aged between 18 and 65 years, diagnosed with a foot-related condition by a qualified healthcare professional, and willing to participate in the study.<br><br>Exclusion criteria will include individuals with insufficient Hindi language proficiency, severe cognitive impairment, medical contraindications that hinder study participation, prior participation in a similar study, concurrent participation in other research studies, and inability to provide informed consent.<br><br>Data Collection and Analysis:<br><br>Once the translated Hindi version of the FFI is developed, a sample of participants will be invited to complete the questionnaire. Data will be collected through face-to-face interviews, ensuring participants’ understanding of the questions and response options. Participants will also have the opportunity to provide feedback on the clarity, relevance, and comprehensibility of the translated FFI.<br><br>Descriptive statistics will be employed to analyze the demographic characteristics of the participants. The comprehensibility and acceptability of the translated FFI will be assessed through qualitative analysis of participants’ feedback, focusing on areas requiring further clarification or

modification. This feedback will inform potential adjustments to improve the clarity and cultural relevance of the Hindi version.

Conclusion:

The pilot study on the cross-cultural adaptation and translation of the Hindi version of the Foot Functional Index aims to provide a culturally adapted tool for assessing foot-related functional limitations and pain in the Hindi-speaking population. By following rigorous translation guidelines and collecting feedback from participants, this study seeks to enhance the comprehensibility and acceptability of the questionnaire, ensuring its accurate application in future research and clinical practice. The findings from this pilot study will serve as a foundation for larger-scale validation studies and contribute to the advancement of cross-cultural research in foot health.
