# Supplementary figures and images for: Cross cultural adaptation and validation of the Hindi version of foot function index
Source: Chiropr Man Therap. 2024 Dec 5;32:38. doi: 10.1186/s12998-024-00563-y (PMC11619674; doi:10.1186/s12998-024-00563-y)

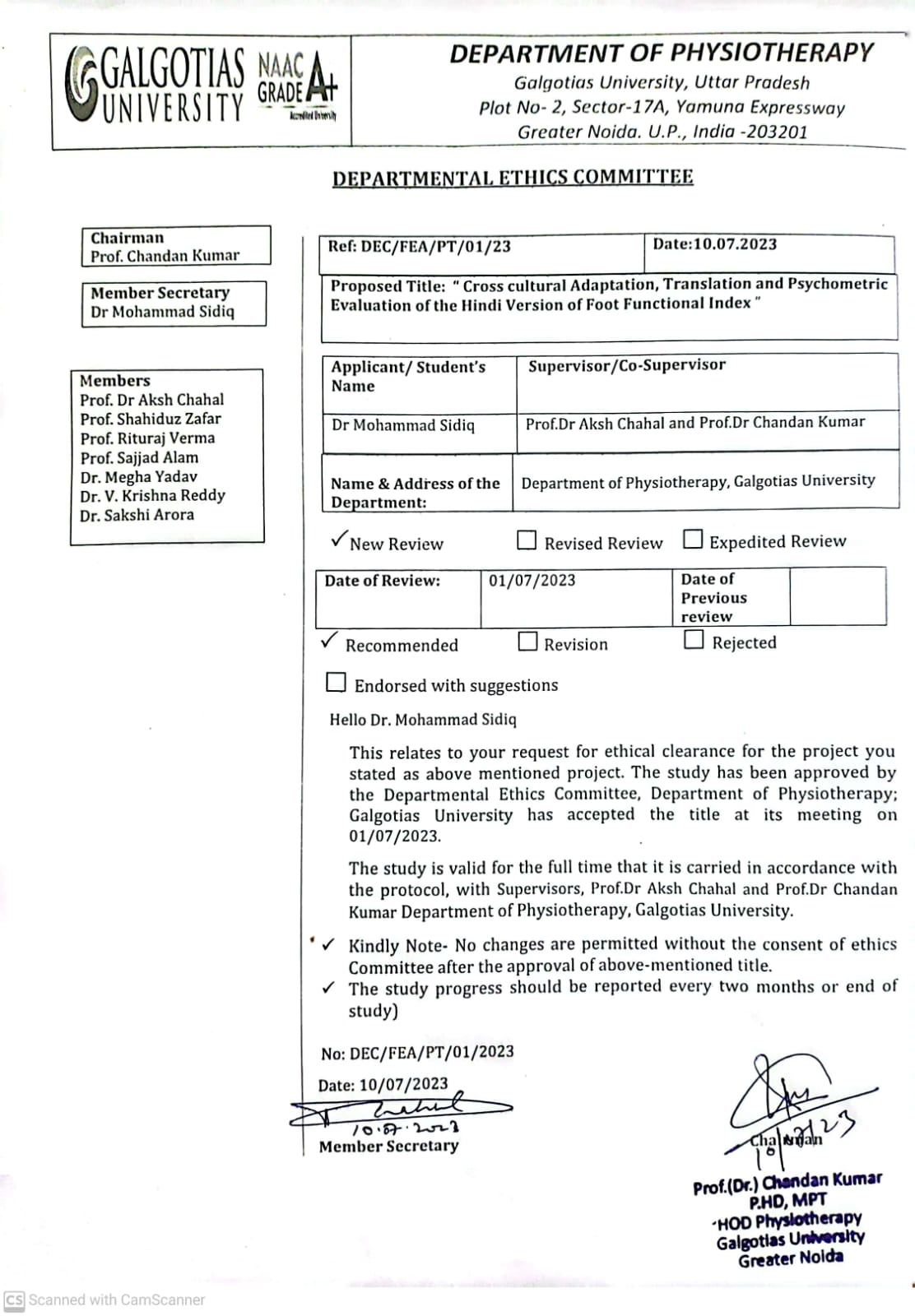

Supplement: Supplementary file 4 — Supplementary Material 4 [file 12998_2024_563_MOESM4_ESM.jpg]
