# Supplementary material for: Cross cultural adaptation and validation of the Hindi version of foot function index
Source: Chiropr Man Therap. 2024 Dec 5;32:38. doi: 10.1186/s12998-024-00563-y (PMC11619674; doi:10.1186/s12998-024-00563-y)
Supplement: Supplementary file 5 — Supplementary Material 5 [file 12998_2024_563_MOESM5_ESM.pdf]

मधुमेह जीवन की गुणवत्ता प्रश्नावली (क्यूओएल-क्यू मधुमेह)©

आपके जीवन की गुणवत्ता

कृपया अभी अपने जीवन के बारे में सोचें। चाहे आपके जीवन की गुणवत्ता अच्छी हो या बुरी, हम फिर भी जानना चाहते हैं। अपने मधुमेह के बारे में सोचें और आपको वर्तमान में क्या करने की आवश्यकता है या क्या नहीं करने की आवश्यकता है । इसे प्रबंधित करने के लिए (उदाहरण के लिए इंसुलिन का इंजेक्शन लगाना या इंसुलिन पंप का उपयोग करना, अपने ग्लूकोज के स्तर की जाँच करना, गोलियाँ लेना, स्वस्थ आहार का पालन करना)।

कृपया प्रत्येक पंक्ति पर एक बॉक्स पर ☑ निशान लगाएं।

क्र.सं.	मेरा मधुमेह और इसे प्रबंधित करने के लिए मुझे क्या करने की आवश्यकता है	पूरी तरह असहमत	असहमत	न तो सहमत, न ही असहमत	सहमत	पूरी तरह सहमत	लागू नहीं
**1**	मैं अपने परिवार/दोस्तों के साथ उस तरह का रिश्ता रख सकता हूं जैसा मैं चाहता हूं, जैसे मिलना-जुलना, आपसी सहयोग, साझा करना रुचियां/अनुभव	☐	☐	☐	☐	☐	☐
**2**	मैं बाहर जा सकता हूं या अपनी इच्छानुसार मेलजोल बढ़ा सकता हूं, उदाहरण के लिए सिनेमा, संगीत कार्यक्रम, दोस्तों के साथ खाना-पीना, व्यस्त या भीड़-भाड़ वाली जगहों पर जाना	☐	☐	☐	☐	☐	☐
**3**	मेरे जैसा रिश्ता हो सकता है। किसी साथी/जीवनसाथी के साथ चाहेंगे । उदाहरण के लिए आपसी सहयोग, हितों को साझा करना /अनुभव	☐	☐	☐	☐	☐	☐
**4**	मैं अपनी इच्छानुसार यौन गतिविधियों का आनंद ले सकता हूँ । जैसे जैसे सहजता, आवृत्ति, क्षमता	☐	☐	☐	☐	☐	☐
**5**	मैं जितना चाहूं शारीरिक रूप से सक्रिय रह सकता हूं, जैसे घूमना, बागवानी, खरीदारी, खेल	☐	☐	☐	☐	☐	☐
**6**	मैं वैसा ही महसूस करता हूं जैसा मैं चाहता हूं, उदाहरण के लिए फिट और स्वस्थ महसूस करता हूं, कोई लक्षण नहीं, स्वस्थ वजन, पर्याप्त ऊर्जा	☐	☐	☐	☐	☐	
**7**	मैं अपने शरीर पर वैसा ही नियंत्रण महसूस करता हूं जैसा मैं चाहता हूं, उदाहरण के लिए कोई ऊंच-नीच नहीं, एक परिवार शुरू कर सकता हूं	☐	☐	☐	☐	☐	
**8**	मैं उतना अच्छा दिखता हूं जितना मैं चाहता हूं, उदाहरण के लिए स्वस्थ दिखूं, जो कपड़े मैं चाहता हूं पहनूं (पंप छुपाने की कोई चिंता नहीं)	☐	☐	☐	☐	☐	
**9**	मुझे अपनी पसंद की छुट्टियाँ मिल सकती हैं जैसे आवास, स्थान, यात्रा	☐	☐	☐	☐	☐	☐
**10**	मैं अपनी इच्छानुसार काम कर सकता हूं, उदाहरण के लिए जिम्मेदारी लेना, पैसा कमाना, अपने करियर में प्रगति करना, पूर्ण	☐	☐	☐	☐	☐	☐

क्यूओएल-क्यू मधुमेह © जेन स्पाइट, 2009. संस्करण 2: संशोधित 24 नवंबर 2016।
उपयोग की अनुमति सहित इस प्रश्नावली के बारे में जानकारी के लिए, कृपया मैपी रिसर्च ट्रस्ट, ल्योन, फ्रांस से संपर्क करें। इंटरनेट: https://eprovide.mapi-trust.org.

	या अंशकालिक						
**11**	मेरे पास बिल, परिवहन लागत, भोजन, उपहार आदि का भुगतान करने के लिए पर्याप्त पैसा है	☐	☐	☐	☐	☐	
**12**	मैं जितना चाहूं गाड़ी चला सकता हूं, उदाहरण के लिए खरीदारी करने जाना, लोगों को लिफ्ट देना, काम पर जाना, अकेले बाहर जाना	☐	☐	☐	☐	☐	☐
**13**	मैं अपने धर्म का पालन/पालन कर सकता हूं । मेरी इच्छानुसार विश्वास, उदाहरण के लिए मेरे पूजा स्थल पर जाना, समुदाय का हिस्सा बनना	☐	☐	☐	☐	☐	☐
**14**	मैं अपनी इच्छानुसार सो सकता हूं, उदाहरण के लिए सो जाओ, सोए रहो, आराम महसूस करो	☐	☐	☐	☐	☐	
**15**	मैं जैसा चाहूं खा सकता हूं, उदाहरण के लिए कब, कहां, क्या, जितना ज्यादा या कम	☐	☐	☐	☐	☐	
**16**	मैं दूसरों की देखभाल कर सकता हूं या उनके लिए उतना उपयोगी हो सकता हूं जितना मैं चाहता हूं जैसे परिवार, दोस्त, सहकर्मी, पालतू जानवर/जानवर, स्वयंसेवा	☐	☐	☐	☐	☐	☐
**17**	मैं अपने पालतू जानवरों/जानवरों के साथ रहने का उतना आनंद ले सकता हूँ जितना मैं चाहता हूँ, उदाहरण के लिए व्यायाम करना, संवारना, खेलना	☐	☐	☐	☐	☐	☐
**18**	मैं जितना चाहूं उतना स्वतंत्र हो सकता हूं, उदाहरण के लिए अपना ख्याल रखना, अकेले बाहर जाना, काम करना या घर पर अकेले रहना	☐	☐	☐	☐	☐	
**19**	मैं अपने जीवन को अपनी इच्छानुसार नियंत्रित कर सकता हूँ जैसे स्वतंत्रता, विकल्प, आगे की योजना बनाना, नियुक्तियाँ रखना	☐	☐	☐	☐	☐	
**20**	मैं जितना चाहूं सहज हो सकता हूं, उदाहरण के लिए बिना किसी सूचना के बाहर जा सकता हूं, जब तक चाहूं बाहर रह सकता हूं	☐	☐	☐	☐	☐	
**21**	मैं "सामान्य" चीजें कर सकता हूं जैसे रोजमर्रा की जिंदगी का आनंद लेना, वह सब करना जो दूसरे लोग बिना किसी चिंता के करते हैं	☐	☐	☐	☐	☐	
**22**	मेरे साथ "सामान्य" व्यवहार किया जाता है, उदाहरण के लिए खुद की देखभाल करने में सक्षम, मेरी उम्र के किसी भी अन्य व्यक्ति की तरह सक्षम	☐	☐	☐	☐	☐	
**23**	मुझमें वह आत्मविश्वास है जो मैं चाहता हूँ, उदाहरण के लिए अकेले या दूसरों के साथ, चुनौतियों का सामना करने में सक्षम	☐	☐	☐	☐	☐	

क्यूओएल-क्यू मधुमेह © जेन स्पाइट, 2009. संस्करण 2: संशोधित 24 नवंबर 2016।
उपयोग की अनुमति सहित इस प्रश्नावली के बारे में जानकारी के लिए, कृपया मैपी रिसर्च ट्रस्ट, ल्योन, फ्रांस से संपर्क करें। इंटरनेट: https://eprovide.mapi-trust.org.
क्यूओएल-क्यू मधुमेह- यूनाइटेड किंगडम/अंग्रेजी - मूल संस्करण
QoL-Q-मधुमेह_AU2.1_eng-GBori

आपके जीवन की गुणवत्ता - महत्व

कृपया अभी अपने जीवन के बारे में सोचें और जीवन का प्रत्येक पहलू आपके लिए कितना महत्वपूर्ण है।

कृपया प्रत्येक पंक्ति पर एक बॉक्स पर ☑ निशान लगाएं।

क्र.सं.		अत्यंत महत्वपूर्ण	महत्वपूर्ण	बिल्कुल नहीं महत्वपूर्ण	लागू नहीं
1	**पारिवारिक रिश्ते/दोस्ती**	☐	☐	☐	☐
2	बाहर जाना या मिलना-जुलना	☐	☐	☐	☐
3	**साथी/पति/पत्नी का रिश्ता**	☐	☐	☐	☐
4	**यौन क्रिया का आनंद लेना**	☐	☐	☐	☐
5	**शारीरिक रूप से सक्रिय रहना**	☐	☐	☐	☐
6	**अच्छा महसूस करना**	☐	☐	☐	
7	अपने शरीर पर नियंत्रण महसूस कर रहा हूँ	☐	☐	☐	
8	**अच्छा लग रहा है**	☐	☐	☐	☐
9	**छुट्टियाँ चल रही हैं**	☐	☐	☐	☐
10	**कार्यरत**	☐	☐	☐	
11	**जो चीज़ें मैं चाहता हूँ उन्हें वहन करना**	☐	☐	☐	
12	**ड्राइविंग**	☐	☐	☐	☐
13	**अपने धर्म का पालन कर रहा हूँ**	☐	☐	☐	☐
14	**सोना**	☐	☐	☐	
15	**जैसा चाहूँ वैसा खाऊँगा**	☐	☐	☐	
16	दूसरों की देखभाल करना या उनके लिए उपयोगी होना	☐	☐	☐	☐
17	पालतू पशु	☐	☐	☐	☐
18	स्वतंत्र रहते हुए	☐	☐	☐	
19	मेरे जीवन पर नियंत्रण होना	☐	☐	☐	
20	सहज होना	☐	☐	☐	
21	"सामान्य" चीजें करना	☐	☐	☐	
22	"सामान्य" के रूप में व्यवहार किया जा रहा है	☐	☐	☐	
23	आत्मविश्वास होना	☐	☐	☐	

धन्यवाद

क्यूओएल-क्यू मधुमेह © जेन स्पाइट, 2009. संस्करण 2: संशोधित 24 नवंबर 2016।
उपयोग की अनुमति सहित इस प्रश्नावली के बारे में जानकारी के लिए, कृपया मैपी रिसर्च ट्रस्ट, ल्योन, फ्रांस से संपर्क करें। इंटरनेट: https://eprovide.mapi-trust.org.
क्यूओएल-क्यू मधुमेह- यूनाइटेड किंगडम/अंग्रेजी - मूल संस्करण
QoL-Q-मधुमेह_AU2.1_eng-GBori
