# Supplementary material for: Cross cultural adaptation and validation of the Hindi version of foot function index
Source: Chiropr Man Therap. 2024 Dec 5;32:38. doi: 10.1186/s12998-024-00563-y (PMC11619674; doi:10.1186/s12998-024-00563-y)
Supplement: Supplementary file 6 — Supplementary Material 6 [file 12998_2024_563_MOESM6_ESM.docx]

**Table 1;** Statistics of participants with painful foot conditions (n = 233) and healthy controls (n = 39)

| Variables | Healthy controls | Participants with painful foot conditions | p |
| --- | --- | --- | --- |
| Age, mean (±) | 49.21 ± 9.8 | 47.1 ± 8.1 | 0.14 |
| Sex |  |  |  |
| Male | 23 (59) | 148 (66.4) |  |
| Female | 16 (41) | 85 (33.6) | 0.58 |
| FFI score, mean (±) | 4.64 ±2.01 | 77.6± 27.11 | < 0.001 |

**Table 2 Parallel analysis:**

**PARALLEL ANALYSIS:**

**Principal Components & Random Normal Data Generation**

Specifications for this Run:

Ncases 223

Nvars 23

Ndatsets 1000

Percentile 95

**Raw Data Eigenvalues, & Mean & Percentile Random Data Eigenvalues**

**Root Raw Data Means Prcntyle**

**1.000000 8.687901 1.632537 1.739545**

**2.000000 1.833182 1.522185 1.600275**

**3.000000 1.568265 1.441517 1.503836**

**4.000000 1.254550 1.372284 1.428869**

**5.000000 1.015277 1.031988 1.039276**

6.000000 .859239 1.253348 1.298999

7.000000 .836943 1.200298 1.244786

8.000000 .739215 1.149707 1.192568

9.000000 .648363 1.101124 1.142149

10.000000 .632678 1.055479 1.096261

11.000000 .604534 1.012737 1.049731

12.000000 .538412 .970250 1.004848

13.000000 .516673 .927313 .965138

14.000000 .475791 .886946 .922289

15.000000 .424572 .846680 .881237

16.000000 .387604 .807997 .842929

17.000000 .374860 .768919 .802673

18.000000 .337521 .729648 .765189

19.000000 .307352 .689153 .726813

20.000000 .290767 .647943 .684167

21.000000 .264376 .606674 .645027

22.000000 .244207 .560930 .602213

23.000000 .157716 .506343 .554625

**Figure 1 Scree plot of parallel analysis (FFI-Hi version, 23 items, n = 223)**


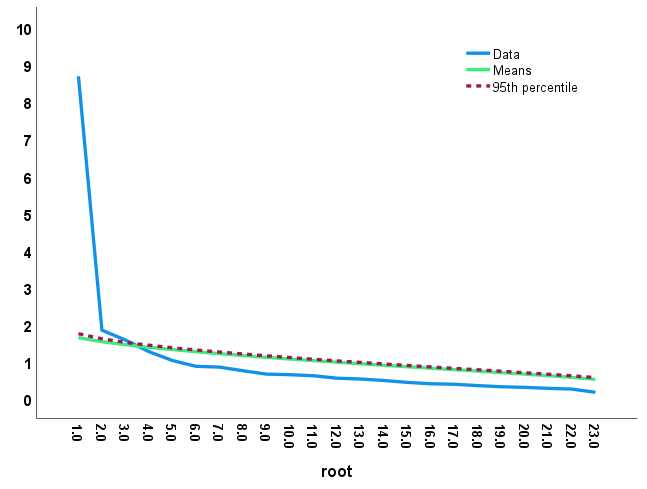


**Table 3 Confirmatory factor analysis**

**Goodness-of-fit**

1. **Chi-square test**

| **Model** | ꭓ^2^ | **df** | **p** |
| --- | --- | --- | --- |
| Baseline model | 2556.13 | 253 |  |
| Factor model | 573.16 | 220 | <0.001 |

1. **Additional fit indices**

| **Index** | **Value** |
| --- | --- |
| Comparative Fit Index (CFI) | 0.867 |
| Tucker-Lewis-Index (TLI) | 0.852 |
| Root mean square error of approximation (RMSEA) | 0.079 |
| Standardized root mean square residual (SRMR) | 0.07801 |
| McDonald fit index (MFI) | 0.432 |
|  |  |

**Table 4a McDonald’s omega coefficient, with an adequate value being ω > 0.80**

| **McDonald’s ω coefficients** | **95% CI** |
| --- | --- |
| 0.91 | 0.90, 0.94 |

**Table 4b McDonald’s omega coefficient ω, if item deleted**

| **Items** | **If item dropped** |
| --- | --- |
|  | **McDonald’s ω** |
| FFI 1 | 0.914 |
| FFI 2 | 0.917 |
| FFI 3 | 0.917 |
| FFI 4 | 0.916 |
| FFI 5 | 0.915 |
| FFI 5 | 0.915 |
| FFI 6 | 0.914 |
| FFI 7 | 0.914 |
| FFI 8 | 0.918 |
| FFI 9 | 0.916 |
| FFI 10 | 0.914 |
| FFI 11 | 0.915 |
| FFI 12 | 0.916 |
| FFI 13 | 0.918 |
| FFI 14 | 0.912 |
| FFI 15 | 0.913 |
| FFI 16 | 0.914 |
| FFI 17 | 0.914 |
| FFI 18 | 0.917 |
| FFI 19 | 0.918 |
| FFI 20 | 0.919 |
| FFI 21 | 0.917 |
| FFI 22 | 0.919 |
| FFI 23 | 0.917 |
